# Early IL-1 receptor blockade in severe inflammatory respiratory failure complicating COVID-19

**DOI:** 10.1073/pnas.2009017117

**Published:** 2020-07-22

**Authors:** Raphaël Cauchois, Marie Koubi, David Delarbre, Cécile Manet, Julien Carvelli, Valery Benjamin Blasco, Rodolphe Jean, Louis Fouche, Charleric Bornet, Vanessa Pauly, Karin Mazodier, Vincent Pestre, Pierre-André Jarrot, Charles A. Dinarello, Gilles Kaplanski

**Affiliations:** ^a^Division of Clinical Immunology, Aix-Marseille Université, Assistance Publique-Hôpitaux de Marseille, 13005 Marseille, France;; ^b^Division of Internal Medicine, L'hôpital d'Instruction des Armées Sainte Anne, 83000 Toulon, France;; ^c^Division of Internal Medicine, Centre Hospitalier Henri Duffaut, 84000 Avignon, France;; ^d^Réanimation des Urgences, Assistance Publique-Hôpitaux de Marseille, 13005 Marseille, France;; ^e^Réanimation Polyvalente des Pathologies du Foie, Assistance Publique-Hôpitaux de Marseille, 13005 Marseille, France;; ^f^Réanimation des Brûlés, Assistance Publique-Hôpitaux de Marseille, 13005 Marseille, France;; ^g^Pharmacy, Assistance Publique-Hôpitaux de Marseille, 13005 Marseille, France;; ^h^Department of Medical Information, Assistance Publique-Hôpitaux de Marseille, 13005 Marseille, France;; ^i^Department of Medicine, University of Colorado, Aurora, CO 80045

**Keywords:** interleukin-1, COVID-19, anakinra, pneumonia

## Abstract

Around the tenth day after diagnosis, ∼20% of patients with coronavirus disease 2019 (COVID-19)−associated pneumonia evolve toward severe oxygen dependence (stage 2b) and acute respiratory distress syndrome (stage 3) associated with systemic inflammation often termed a “cytokine storm.” Because interleukin-1 (IL-1) blocks the production of IL-6 and other proinflammatory cytokines, we treated COVID-19 patients early in the disease with the IL-1 receptor antagonist, anakinra. We retrospectively compared 22 patients from three different centers in France with stages 2b and 3 COVID-19−associated pneumonia presenting with acute severe respiratory failure and systemic inflammation who received either standard-of-care treatment alone (10 patients) or combined with intravenous anakinra (12 patients). Treatment started at 300 mg⋅d^−1^ for 5 d, then tapered with lower dosing over 3 d. Both populations were comparable for age, comorbidities, clinical stage, and elevated biomarkers of systemic inflammation. All of the patients treated with anakinra improved clinically (*P* < 0.01), with no deaths, significant decreases in oxygen requirements (*P* < 0.05), and more days without invasive mechanical ventilation (*P* < 0.06), compared with the control group. The effect of anakinra was rapid, as judged by significant decrease of fever and C-reactive protein at day 3. A mean total dose of 1,950 mg was infused with no adverse side effects or bacterial infection. We conclude that early blockade of the IL-1 receptor is therapeutic in acute hyperinflammatory respiratory failure in COVID-19 patients.

Coronavirus disease 2019 (COVID-19), the severe acute respiratory syndrome coronavirus 2 (SARS-CoV-2)−associated disease, can be classified into three clinical stages. In stage 1, an estimated 80 to 84% of infected patients are slightly symptomatic. In stage 2a, patients have a nonhypoxemic pneumonia but can advance to a hypoxemic pneumonia (stage 2b) or acute respiratory distress syndrome in stage 3 (1, 2). After ∼9 d to 10 d, 17 to 20% of patients can evolve toward more severe stages 2b or 3, with increasing requirement for oxygen necessitating admission into intensive care units (ICU) with noninvasive or invasive mechanical ventilation (IMV) (1, 2). This progression of the disease is associated with high levels of circulating proinflammatory cytokines, often termed a “cytokine storm” (3).

SARS-CoV-2 activates the NLRP3 inflammasome, resulting in the processing and secretion of active IL-1β and IL-18 and the initiation of a cytokine release syndrome (4, 5). IL-1β induces IL-6 and a Th-17 immune response (5), whereas IL-18 induces IFN gamma (IFNγ) production by Th-1 lymphocytes (6). An IL-1/IL-6 signature increases neutrophils and C-reactive protein (CRP), whereas an IL-18/IFNγ signature is characterized by hyperferritinemia and cytopenia (7). A majority of patients with COVID-19 exhibit a predominant IL-1/IL-6 signature, but can evolve into an IL-18/IFNγ signature mimicking cytokine profiles observed in other inflammatory diseases such as macrophage activation syndrome (MAS) (8). Anakinra is highly effective in MAS (8) and significantly decreased all-cause mortality (9). We thus hypothesized that blocking IL-1 early using anakinra is a therapeutic option in patients with severe stage 2b and early stage 3 COVID-19.

## Patients and Results

### Study Design and Patients.

This study was approved (INDS, MR4115050520) by the Assistance Publique-Hôpitaux de Marseille for compassionate use; participants receiving anakinra gave written informed consent. COVID-19 pneumonia was diagnosed in patients with a positive PCR for SARS-CoV-2 in nasopharyngeal samples, respiratory symptoms, and a concordant pneumonia on low-dose computed tomography (CT) scan, scored from 0 to 5 according to the severity (10). Between the 5th and 13th days of the diagnosis, the patients presented severe hypoxia requiring oxygen therapy and were classified as stage 2b or 3, according to the 2020 clinical staging proposal (2). Anakinra was started with a rapidly deteriorating condition consisting of increased oxygen requirement of >4 L/min within the previous 12 h, and CRP above 110 mg/L with or without fever higher than 38.5 °C.

**Fig. 1.**
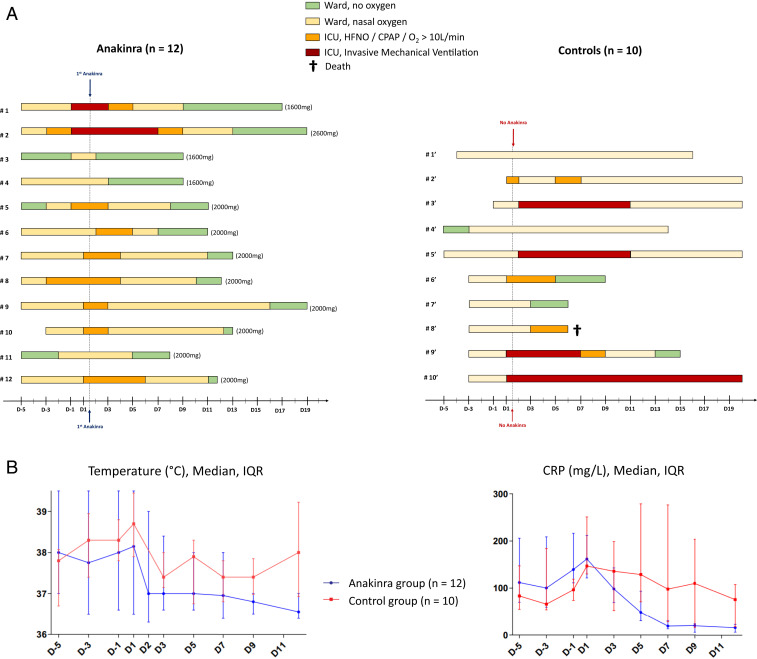
(*A*) Daily disposition of the 12 patients treated with anakinra and the 10 control patients treated with standard of care only; the total dose of anakinra for each patient is indicated in parentheses. (*B*) Body temperatures (degrees Celsius), and CRP (milligrams per liter) evolution over time, in patients who received anakinra or in controls. IQR, interquartile range.

A control group of patients were selected among 30 patients seen in Avignon and Toulon to closely match the inclusion criteria for treatment with anakinra (Dataset S1). The control group standard of care included antibiotics and hydroxychloroquine, and two patients in Avignon received ritonavir/lopinovir. Anakinra-treated patients received no antivirals. Day 1 (Fig. 1) corresponds to anakinra initiation and the day the control group fulfilled the inclusion criteria defined for the anakinra group. Anakinra (Swedish Orphan Biovitrum) was infused intravenously (i.v.) over 2 h as a single daily dose of 300 mg for 5 d, then tapered to 200 mg⋅d^−1^ for 2 d, and then 100 mg for 1 d. Also at this time, all patients but one had at least one negative PCR test for SARS-CoV-2 in nasopharyngeal samples. Patients’ clinical outcome follow-up was recorded after 20 d. Dataset S1 details statistical analyses of the data and comparison with other studies using anakinra.

Twelve patients received anakinra, and 10 were in the control group. The groups were comparable in terms of age, gender, comorbidities, and the severity of the disease, as judged by the NEWS score at day 1. Seventy-two percent of patients in both groups were febrile, with systemic inflammation as judged by elevated CRP and ferritin concentrations (Dataset S1). Initial lung CT scans showed moderate to severe pneumonia (grade 3). Among the 10 patients from the control group, 4 required IMV, 2 required high-flow nasal oxygen (HFNO) in the ICU, and 4 patients in the wards required nasal oxygen flow of >10 L/min. One patient died, one patient was still intubated after day 20, and the 4 others left the ICU, with a mean of 9.5 d. Among the 12 patients who received anakinra, 2 required IMV, 6 required HFNO or continuous positive airway pressure (CPAP) in the ICU, and 4 required nasal oxygen flow of >10 L/min in the wards. All of the patients treated with anakinra survived and were discharged from the ICU, with a mean of 5 d (Fig. 1*A*).

Clinical improvement consisting of the NEWS score at day 5 (*P* < 0.01; Dataset S1) and the number of days with oxygen flow less than 3 L/min at day 20 (*P* < 0.05) was observed in the patients who received anakinra. Although the numbers of days without IMV was higher in the anakinra group, the difference did not reach statistical significance (*P* < 0.06). The response to anakinra was rapid, with reduction of fever within 48 h (*P* < 0.05; Fig. 1*B*), whereas fever persisted in some control patients despite allowance of paracetamol. There was rapid reduction of CRP in the anakinra group; the log-linear mixed model yielded a significant interaction between day and group (*P* < 0.001), showing that evolution of CRP was different between the two groups.

To compare groups at baseline and at particular end points, we used Fisher’s exact test for qualitative variables and the nonparametric Mann−Whitney test for quantitative variables (two-sided, alpha error = 0.05). Qualitative variables are expressed using percentages, and quantitative variables are expressed using median and interquartile range. To analyze the evolution of CRP and temperature at different end points (days 1, 3, 5, 7, and 9), we performed two univariate mixed model (with log-linear distribution), with random intercept and group and interaction between groups and day as fixed effects.

## Discussion

The clinical course of severe COVID-19 patients is the rapid deterioration of respiratory function associated with rising markers of systemic inflammation (CRP and ferritin). As we report here, clinical and biomarker rescue took place within 24 h to 48 h of the first dose of anakinra. Anakinra is a highly effective receptor antagonist because its affinity for IL-1R1 is greater than that for IL-1 itself (5). IL-1Ra prevents the inflammation driven by either IL-1α and IL-1β, and is approved to treat rheumatoid arthritis for subcutaneous (s.c.) administration of 100 mg daily, but is widely used off-label (11). Anakinra is also used i.v. to treat MAS, associated with a severe cytokine storm (12).

Stages 2b and 3 COVID-19 are characterized by high circulating levels of inflammatory cytokines, notably IL-6 (3). Although the anti-IL-6 receptor monoclonal antibody, tocilizumab, has been used to treat COVID-19 patients (13, 14), any and all therapies that reduce the activities of either IL-1β or IL-1α consistently reduce circulating levels of IL-6 (5, 11). Because of the rapidity of action and the short half-life, we treated our patients with i.v. anakinra at 300 mg⋅d^−1^, a dose threefold higher than the approved s.c. dose of 100 mg, but five times lower than doses used to treat MAS (9, 12). Importantly, anakinra was started early in acute respiratory failure in order to arrest deterioration and reduce the need for IMV. With a total dose under 2,000 mg, anakinra resulted in a rapid fall in fever and CRP, reduced oxygen requirements, and led to fewer days in the ICU without IMV, compared to the control group. A study in 30 Italian COVID-19 patients reports efficacy and decreased deaths with anakinra in more severely affected patients, but required higher doses (10 mg⋅kg^−1^⋅d^−1^) for a longer period of time (15). In the present study, a lower dose of 4 mg⋅kg^−1^ was administered for only 5 d, followed by a rapid tapering. Early use of anakinra may be an important clinical decision when respiratory conditions begin to deteriorate in the context of increasing systemic inflammation.

This report has limitations, such as the low number of patients and being a retrospective study. A prospective randomized, placebo-controlled study of anakinra in COVID-19 is planned by the French authorities (NCT04366232).

### Data Deposition.

All study data are included in the article and Dataset S1.

## Supplementary Material

Supplementary File
