# Supplementary material for: Early IL-1 receptor blockade in severe inflammatory respiratory failure complicating COVID-19
Source: Proc Natl Acad Sci U S A. 2020 Jul 22;117(32):18951–3. doi: 10.1073/pnas.2009017117 (PMC7430998; doi:10.1073/pnas.2009017117)
Supplement: Supplementary File [file pnas.2009017117.sd01.pdf]

Data Set S1 Patients Characteristics and Outcomes

Patients	Anakinra (n = 12)	Control (n = 10)	*P* value
**Demographics**			
**Male / Female**, n (%)	6 (50%) / 6 (50%)	6 (60%) / 4 (40%)	0.69
**Age** (years), Median [IQR]	61 [54-68]	58 [45-69]	0.61
**BMI** (kg/m^2), Median [IQR]	25.3 [24.6-30.9]	27.2 [26.2-28.9]	0.96
**Comorbidity,** n (%)			
- High Blood Pressure	4 (33.3%)	3 (30%)	0.99
- Cardiovascular disease	1 (8.3%)	2 (20%)	0.57
- Diabetes	0 (0%)	3 (30%)	0.08
- Chronic Respiratory disease	2 (16.7%)	4 (40%)	0.35
- Tobacco smoking	3 (25%)	1 (10%)	0.63
- Chronic kidney disease	0 (0%)	1 (10%)	0.45
**Clinical and Paraclinical parameters at Day 1**			
- Temperature (°C), Median [IQR]	38.2 [37.6-38.5]	38.7 [37.9-39.5]	0.09
- NEWS score (severity), Median [IQR]	8 [7.25-9]	7.5 [5.75-10.25]	0.46
- CRP (mg/L), Median [IQR]	162 [122-212]	147 [130-251]	0.58
- Ferritin (µg/L), Median [IQR]	1797 [972-3119]	1165 [1019-3706]	0.87
- CT-scan severity, Median [IQR]	3 [2-3]	3 [2-3]	0.84
- Patients with invasive ventilation at Day 1, n (%)	2 (17%)	2 (20%)	0.99
**Outcomes (20 days follow-up)**			
- NEWS score (severity) at Day 5, Median [IQR]	4.5 [2-5]	8 [6.5-11.25]	< 0.01
- Number of days with O_2 < 3L/min, Median [IQR]	15.5 [13.25-17.5]	8 [0 - 15]	< 0.05
- Number of ICU free days, Median [IQR]	17.5 [16.25-20]	14 [9.5-20]	0.17
- Number of Invasive ventilation free days, Median [IQR]	20 [20-20]	17 [9.5-20]	0.06
- Death, n (%)	0 (0%)	1 (10%)	0.45

www.pnas.org/cgi/doi/10.1073/pnas.2009017117
